# Feasibility of attention-based virtual reality interventions in fibromyalgia syndrome: comparing systems, virtual environments and activities

**DOI:** 10.1177/20494637241310696

**Published:** 2025-01-04

**Authors:** Jordan Tsigarides, Vanessa Grove, Jacqueline Chipping, Jack Dainty, Susan Miles, Nicholas Shenker, Saber Sami, Alexander Macgregor

**Affiliations:** 1 Faculty of Medicine and Health Sciences, Norwich Medical School, 83726 University of East Anglia, Norwich, UK; 2 Department of Rheumatology, 89744 Addenbrookes University Hospital, Cambridge, UK

**Keywords:** Chronic pain, fibromyalgia, pain management, virtual reality intervention, feasibility

## Abstract

**Background:**

Fibromyalgia Syndrome (FMS) is highly prevalent with a significant associated morbidity and socioeconomic burden. Effective treatments for FMS remain elusive with pharmacological management (including use of opioids) often proving ineffective. There is a need to develop accessible, innovative management approaches to improve patient care.

Virtual reality (VR) interventions have shown evidence of efficacy in the management of acute pain and chronic low back pain, but their feasibility in FMS has not hitherto been explored.

**Methods:**

This feasibility study investigates the use of four different VR systems, four interactive VR activities and two virtual environments in patients with FMS. Acceptability (including adverse effects) and study engagement were the main outcomes investigated. Clinical outcome data on pain and mood were also collected to gather preliminary information for future studies.

**Results:**

The results demonstrated good feasibility across VR systems, activities and virtual environments with high levels of acceptability, low frequency of adverse effects, and positive perceptions of VR in patients with FMS. Reporting of adverse effects (including fatigue) varied across different VR components, with system comfort and virtual environmental design being particularly important.

**Conclusions:**

The findings increase our confidence with respect to the feasibility of using VR in people with FMS, help to inform future randomised controlled trials and emphasise the importance of tailored interventional design for future VR therapeutics.

## Introduction

Fibromyalgia Syndrome (FMS) affects between 4 and 6% of the adult population^1,2^ and is characterised by chronic widespread pain, fatigue, sleep disturbance, cognitive ‘fog’, and significant psychological distress.^3^ Despite the socioeconomic burden and associated morbidity, effective treatments for FMS remain elusive with pharmacological management (including use of opioids) often proving ineffective.^4^ The lack of pharmacological efficacy has led to the European League Against Rheumatism (EULAR) and National Institute of for Health and Care Excellence (NICE) guidance recommending non-pharmacological approaches as first-line.^5,6^ However, the accessibility and acceptability of recommended therapeutic options including supervised aerobic exercise can be challenging due to factors including resource constraints and psychological factors.^7^ There is a need for novel, accessible, and well-tolerated therapeutic strategies for this patient population.

Virtual Reality (VR) has attracted increasing interest in pain management given evidence of analgesic efficacy in acute pain.^8,9^ This novel technology immerses users within a fully interactive, computer-generated virtual environment through use of a head-mounted display (HMD). The immersion in VR is achieved by fully engaging the user’s visual and auditory senses and effectively removing distractions from the outside world. Pain demands attention^10^ and the immersive nature of VR has great potential in re-allocating attentional resources away from pain (also known as attentional modulation) and supporting personalised management approaches.^11,12^

However, VR interventions are not uniform; they consist of multiple modifiable parts including hardware, virtual environments and activities. These components can significantly influence the overall experience and efficacy of VR as a pain management tool.^13–15^ Small feasibility studies investigating attention-based VR (interventions primarily aimed at engaging attention through VR activities) in heterogenous chronic pain cohorts have shown promising results. These interventions have only included single activities completed within one virtual environment (often using a ‘rail-shooter’ game mechanic within a cold virtual environment as investigated within the acute pain literature).^16,17^

When evaluating VR in chronic pain, the challenges of specific patient groups need consideration to avoid a potentially inappropriate ‘one-size-fits-all’ approach. This is particularly important in FMS, given those living with the condition face specific challenges including severe fatigue, sensory hypersensitivity (including sound, light and temperature), and cognitive symptoms^18^ that will inevitably influence their experience of VR interventions. Despite highly promising results from recent RCTs investigating VR in chronic low back pain,^19,20^ there are no studies that have specifically investigated the feasibility of different VR head-mounted systems (with different technical capabilities), interactive activities (with different mechanics), and virtual environments in FMS.

To address the lack of VR research conducted in FMS cohorts, this feasibility study’s primary goal was to determine the acceptability of different VR system configurations, interactive activities, and virtual environments. This work is essential to ensure that subsequent larger studies, designed to rigorously test the efficacy of VR pain interventions in FMS, are built on a solid foundation of feasibility and patient acceptability data.

## Methods

### Study design and ethics

This study was designed as a feasibility study aiming to investigate the acceptability of different (1) VR systems, (2) Virtual environments, and (3) interactive VR activities in patients with FMS. This conforms to a ‘VR2’ design as outlined by the VR-CORE international working group.^21^ Data collection sessions (i.e. sessions in which participants completed the interventions) were conducted within an allocated room at Norwich Medical School, with adjustments made to the data collection protocol to account for safety considerations during the COVID-19 pandemic. This study was approved by the Cornwall and Plymouth Research Ethics Committee (REC Reference 20/SW/0050) and registered with the International Standard Randomised Controlled Trial Number registry (ISRCTN46681140).

### Participants and screening

Recruitment took place from October 2020 to April 2021. Participants were directly identified by clinicians within general rheumatology outpatient clinics at the Norfolk and Norwich University Hospital and Addenbrookes Hospital (Cambridge). Recruitment sites were chosen based on locality to the study site for the convenience of researchers and participants. The inclusion criteria were (1) adults ≥18 years of age; (2) ability to understand and speak conversational English; (3) capacity to consent; (4) diagnosis of FMS fulfilling the 2016 diagnostic criteria.^18^ Exclusion criteria were (1) presence of co-morbid condition(s) exacerbated by exposure to flashing lights or screens; (2) a diagnosis of cognitive impairment; (3) current visual or hearing impairment that would prevent the participant from using the technology included the research.

### Procedures

#### Overall study flow

**Figure 1.**
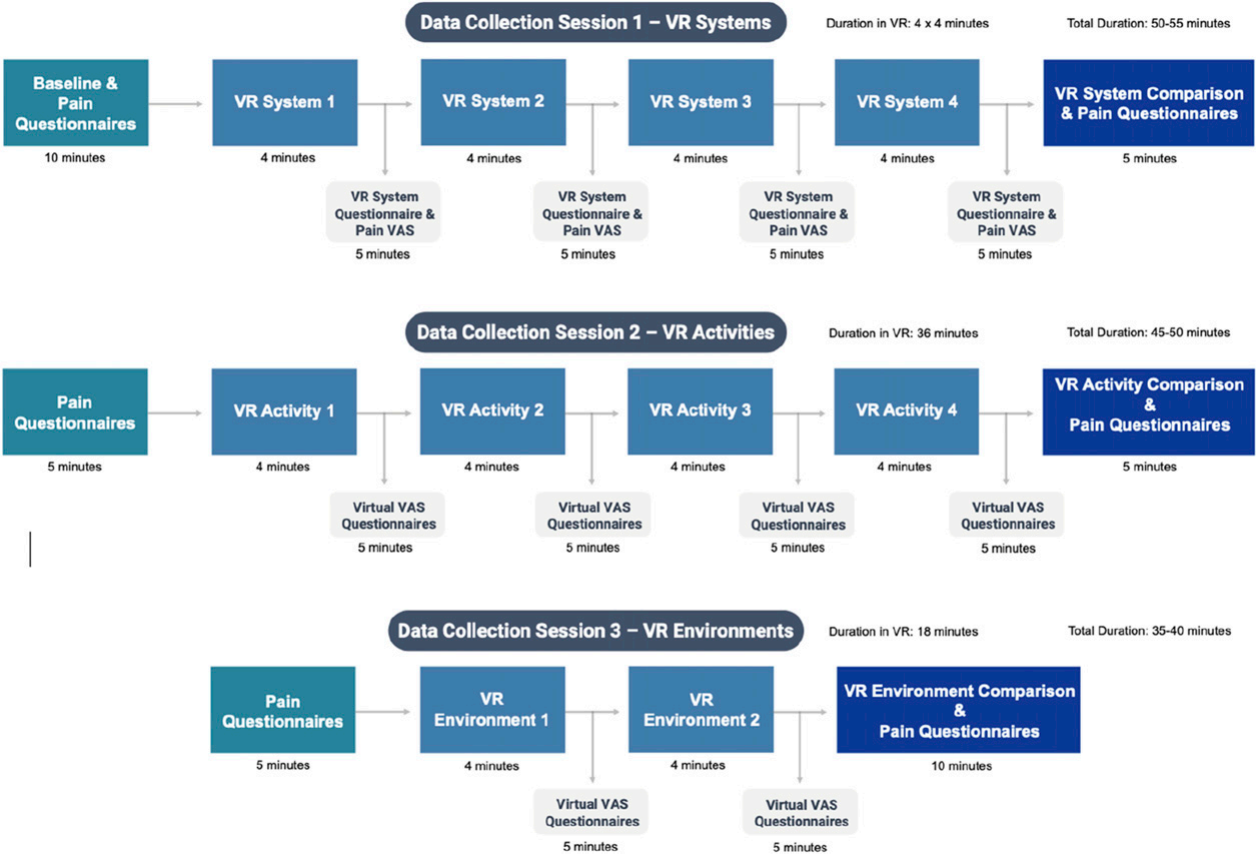
Flow diagram showing the steps of each data collection session, durations of each step, and total durations.

The overall study flow is outlined in Figure 4, with the stages in each data collection session outlined in Figure 1. Following identification, individuals were given an invitation letter and participant information sheet clearly explaining the study and stating the inclusion and exclusion criteria. Those wishing to participate returned a reply slip contained within the invitation letter or contacted the research team via email or telephone to express their interest. Participants were then screened via telephone by a member of the research team and invited to data collection sessions. Each of the three data collection sessions investigated a different component of the VR experience:

- Data Collection Session 1: Four different VR systems
- Data Collection Session 2: Four different VR activities
- Data Collection Session 3: Two different VR environments

To minimise carryover effects, there was a mandatory ‘washout’ period of at least 1 week between sessions. One week has been used as a washout period in other VR studies that investigated similar interventions.^22^ Within each session, the sequence of interventions was determined using simple randomisation, employing random number tables to ensure an unbiased distribution of the order in which interventions were experienced.

Upon attending the first data collection session, participants completed a written consent form and baseline questionnaires including demographics, disease severity (including pain severity), co-morbidities and previous exposure to VR. Capacity for participants to provide informed consent for the procedures included in the study was assessed by the research team at the time of consent form completion and reassessed at subsequent data collection sessions. Ability to read and understand written English questionnaires was checked with each participant by a member of the study team. No adaptations to procedures or technologies included within data collection sessions were required to allow participants to take part.

#### Facets of feasibility tested

Acceptability (including adverse effects) and study engagement were the main outcomes investigated in this study. Acceptability pertains to the degree in which participants found VR interventions engaging, immersive, usable, comfortable and satisfactory overall. Adverse effects are defined as any unfavourable or harmful events that occur during or after the VR interventions and are reasonably associated with their use (such as physical discomfort). Study engagement relates to participants’ willingness to participate (including rates of study recruitment and retention), and their perceptions of VR after use. Feasibility encapsulates all of the above, and provides an overall indicator of the practicality and potential for successful implementation of the VR interventions in larger-scale studies.^23^ Clinical outcome data on pain and mood were also collected to gather preliminary information for future larger-scale studies.

#### The VR application development and content

**Figure 2.**
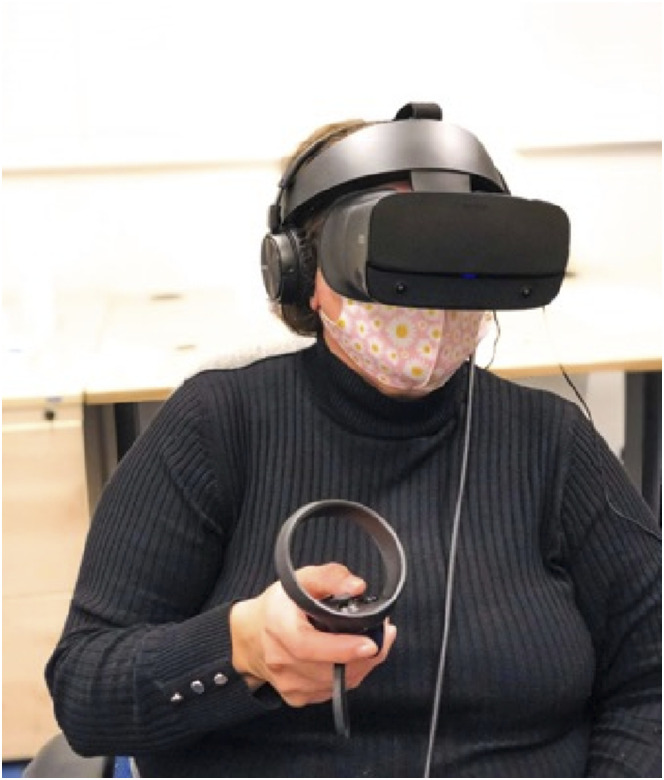
Participant seated, using the Oculus Rift S during Data Collection Session 1.

The VR application was developed specifically for the purposes of this research alongside industry partners (Orbital Global Ltd) with input from the research team comprising of academic clinicians and researchers with expertise in chronic pain, neuroscience, virtual reality, game design and psychology. The application was designed specifically for patients with chronic pain with careful consideration of the audio-visual experience, activity-related movement requirements, and orientation of the experiences. VR environments were set within a tranquil forest, accompanied by relaxing sounds to create a serene atmosphere for participants during the activities. Similar forest settings have been used to promote relaxation in previous VR studies such as the ‘Virtual Meditative Walk’.^24^ Over-ear headphones were used to optimise audio quality and promote immersion, with participants always remaining seated during VR use (although able to stand up and move around when not using VR). Hand-held controllers were used to enable interaction (Figure 2). A starting area with a selection menu allowed the user to choose from four activities and two virtual environments. This starting area also included a VR questionnaire that enabled collection of virtual visual analogue scale (VAS) data for evaluation of activities and virtual environments in Data Collection Sessions 2 and 3. Additional details are provided in Appendix 1.

#### Data collection session 1: The VR systems

Four head-mounted VR systems were investigated, each with different characteristics representing the spectrum of commercially available technology at the time. Systems included the Samsung Gear VR (Samsung Electronics, Ridgefield Park, New Jersey) with a Samsung Galaxy S8 smartphone, Oculus Go, Oculus Quest and Oculus Rift S (Reality Labs, Menlo Park, California). One hand-held controller specific to each VR system was used to enable interaction (e.g. Gear VR controller, Oculus Go controller, Oculus Quest and Rift touch controllers). See the Appendix for a comparison of technical specifications. Custom head mounting straps were used with the Oculus Go and disposable fascia covers used with the Samsung Gear VR to ensure adequate infection control procedures between users.

Participants used each system in a random order, experiencing the same interactive VR activity (rail-shooter activity) within the same virtual environment (cold) with each system. See Figure 1 for the durations of each step in this session, and sections describing data collection sessions 2 and 3 below for descriptions of the rail-shooter activity and virtual environments.

#### Data collection session 2: The VR activities

Four time-restricted VR activities were created, each requiring the user to utilise skills in attention but with each challenging further skills through different game ‘mechanics’ (Table 1).

**Table 1.** Comparison of the VR activities investigated in Data Collection Session 2.

Activity type	Main skills required	Short activity description	Image
Rail-shooter	Hand-eye co-ordination and agility	To aim at and shoot targets appearing in the sky whilst travelling steadily along a calm river	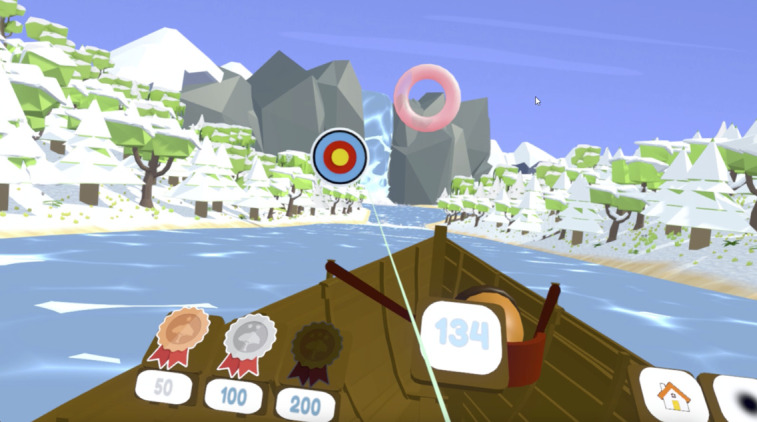
Memory	Short-term and working memory	To recall sequences of objects emerging from plots within a 3x3 grid. Upon correct recall, sequences increased in length	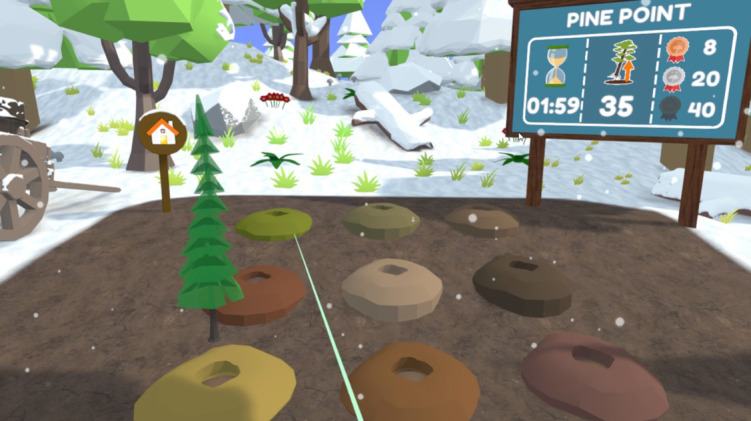
Multitasking	Strategic planning and task prioritisation	To follow a series of steps to plant, maintain and harvest trees across five plots simultaneously	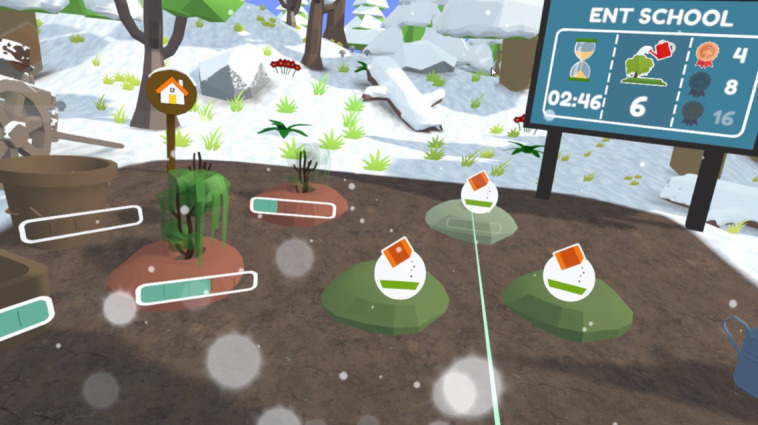
Match-3	Pattern recognition, visuospatial awareness and strategic planning	To match three of the same objects in a row across three vines by moving vines up or down	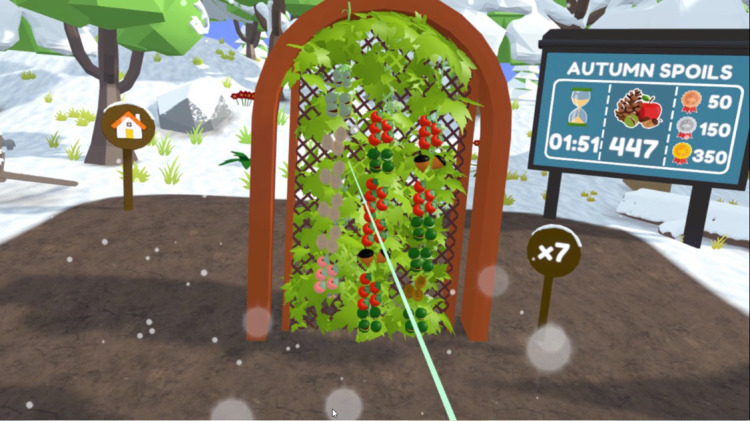

Elements of ‘gamification’ were added to each of the activities to encourage engagement, motivation and entry into a psychological state of ‘flow’ which is thought to promote task focus with increased attentional modulation.^25^ This included a scoring system dependent on performance, with scores displayed to the user during the activity. The types of activities included were carefully selected for the following reasons:

(1) Each activity type challenges different cognitive skills (as outlined in Table 1) – Many of which can be impaired in FMS (e.g. short-term memory).
(2) Activity types included (rail-shooter, memory, multitasking, match-3) are commonly used in other contexts as cognitively engaging, attention-demanding tasks.
(3) Previous VR pain research have demonstrated immersion, engagement and pain relief in similar tasks.^9,16,17,19^
(4) Each activity used the same mode of interaction (controller-based), with a similar intensity of interaction, and a similar low intensity of required movement (both the arms and head). This was to ensure an even level of physical activity intensity across the activities, and to reduce risk of pain exacerbation in FMS participants that commonly suffer with pain affecting the head, neck and arms.

Participants used the Rift S VR system (with rift touch controller) to complete each of the four VR activities within the same virtual environment (cold). The order in which they experienced the activities was random. See Figure 1 for the durations of each step in this session.

#### Data collection session 3: The virtual environments

**Figure 3.**
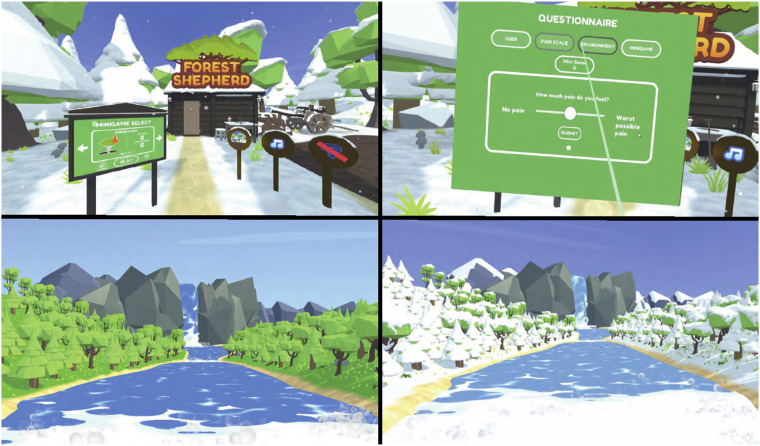
Images of the VR application used. Top left: Starting menu area; Top right: VR data collection questionnaire with virtual VAS; Bottom left and right: Cold and warm environments respectively.

Two contrasting virtual environments were investigated, one simulating a cold (snowy) world and another a warm (sunny) world within VR (Figure 3). These contrasting virtual environments were designed based on previous acute pain VR research that utilised virtual environments depicting winter scenes^26^ and evidence of thermodysregulation states in FMS.^27^ Each virtual environment provided a different audio-visual experience but did not alter the functionality or interactivity of the VR program. Participants used the Rift S VR system (with rift touch controller) to complete the same VR activity (rail-shooter) within each of the two virtual environments. The order in which they experienced the virtual environments was random. See Figure 1 for the durations of each step in this session.

### Measures

All measures were delivered in English and completed during the lab-based data collection sessions with no longitudinal follow-up given the feasibility nature of the study. Table 2 provides a summary of measures used. The Appendix provides more detailed information on each measure used including the calculation method for composite scores for each outcome.

**Table 2.** A summary table describing the evaluation instruments used and their frequency of administration.

Questionnaire	Frequency of administration	Session used	Description
*Widespread Pain Index (WPI) & Symptom Severity Scale (SSS) Score* ^18^	Baseline only	Session 1	To measure Fibromyalgia Severity (FS) and ensure participants fulfil 2016 FMS diagnostic criteria
*Revised Fibromyalgia Impact Questionnaire (FIQR)* ^28^	Baseline only	Session 1	To determine the impact of FMS on activities of daily living, overall wellbeing and severity of symptoms
*Patient Health Questionnaire-9 (PHQ-9)* ^29^	Baseline only	Session 1	To determine presence and severity of depressive symptoms
*Generalised Anxiety Disorder Assessment 7 (GAD-7)* ^30^	Baseline only	Session 1	To determine presence and severity of anxiety symptoms
*McGill Pain Questionnaire – Short Form* ^31^	Pre- and post-intervention	All	To understand baseline and the changes in self-reported pain severity and character
*Visual Analogue Scale – Pain*	Pre- and post-intervention	All	To understand baseline and the changes in self-reported pain severity
*Subjective experience questionnaires*	Post-intervention	All	Specific questionnaires for each session used to gain information on acceptability, feasibility, tolerability and perceptions of VR following interventions
*Virtual Reality Sickness Questionnaire (VRSQ)* ^32^	Post-intervention	All	To quantify adverse effects experienced with interventions
*Virtual Visual Analogue Scale – Enjoyment, engagement, difficulty, length of time, motivation to perform well related to VR activity (Virtual VAS)*	Post-intervention	Session 2	To quantify participant experience of different VR activities
*Virtual Visual Analogue Scale – Enjoyment, immersion, feelings of stress, changes in mood related to virtual environment*	Post-intervention	Session 3	To quantify participant experience of different virtual environments

#### Acceptability – User experience, usability, overall comfort and adverse effects

Post-intervention self-report questionnaires were used to gain information on the overall comfort, usability and immersive nature of VR. Written questionnaires posed a combination of rating style (1-7 scale), multiple choice, and white space questions. Rating question data were dichotomised; participants reporting a rating of 5-7 were categorised as agreeing with the statement and participants reporting 1-3 as disagreeing. Virtual questionnaires (completed in VR) gained VAS ratings specific to the VR activity or virtual environment experienced.

The Virtual Reality Sickness Questionnaire (VRSQ)^32^ was used post-intervention to collect data on adverse effects. The VRSQ is a validated tool designed to assess the physical discomfort experienced with VR interventions and has been shown to have better psychometric qualities for assessing VR applications than the traditionally used Simulator Sickness Questionnaire (SSQ).^33^ The VRSQ includes a list of nine symptoms observed with use of VR systems, each scored based on severity (0-3, none to severe), with a score range of 0-100.

#### Study engagement and perceptions

Rating questions within the self-report post-intervention questionnaires were used to understand participants’ perception of VR following each intervention. This included questions enquiring about their openness to using VR at home for pain management. In addition, data on participant recruitment and retention rates were recorded.

#### Clinical outcomes

The McGill Pain Questionnaire Short Form (MPQ-SF),^31^ pain VAS, and specific questions within subjective experience questionnaires were used to gain data on pain intensity and quality, as well as changes in pain perception over the course of the study and across different interventions. The MPQ-SF and written pain VAS were completed at the start of each data collection session prior to VR use. The MPQ-SF was completed again at the end of sessions after completing interventions. A written pain VAS was completed immediately after use of each VR system within Data Collection Session 1. A virtual pain VAS was completed immediately after experiencing each VR activity or environment in Data Collection Sessions 2 and 3 respectively, enabling participants to report pain intensity without needing to remove VR systems. Both VAS measures included a continuous horizontal scale in which participants marked their pain intensity at the time of measurement from ‘No Pain’ to ‘Worst Possible Pain’. The horizontal line for the written VAS was 10 cm in length and an equivalent non-numerical scale was used for the virtual VAS. See the Appendix for more detailed information on clinical outcome instruments used.

At baseline, two validated scales were used to measure symptoms of depression and anxiety: the Patient Health Questionnaire-9 (PHQ-9)^29^ and the Generalised Anxiety Disorder 7-item scale (GAD-7).^30^

Measures of mood and subjective stressfulness of each of the two virtual environments were used, with participants completing a blinded virtual VAS for ‘feelings of stress’ (0 least stressed, 10 most stressed) and ‘changes in mood’ (0 worsened mood, 10 improved mood) within session 3. Multiple choice and white space questions were included within subjective experience questionnaires in sessions 2 and 3 to understand post-intervention changes in mood.

### Data analysis

Statistical analyses were conducted in RStudio 2022.02.1 Build 461. Given the feasibility nature of this study, sample size was not calculated based on efficacy outcomes. A target of between 20 and 30 participants for recruitment was set as this sample range is large enough to enable meaningful qualitative interpretation and to detect obvious flaws in the study design, yet resource-efficient considering the nature of the investigation.

Descriptive statistics were used to summarise demographic data. Normality of continuous data was assessed using the Shapiro-Wilk test. Categorical data were reported as absolute numbers, while continuous data were reported as means, standard deviations, and ranges where appropriate. Changes in means with 95% confidence intervals (to provide an estimate of the precision of the findings) were reported for pain outcome data within the Appendix. Data from 7-point rating scale questions were dichotomised, with scores of 5-7 categorised as agreement with the statement, and scores of 1-3 as disagreement.

For normally distributed data, t-tests were used, such as in comparing acceptability rating total scores across the two virtual environments (warm vs cold) in Data Collection Session 3. For non-normally distributed data, a Wilcoxon Signed Rank test was used for pairwise comparisons, including the VRSQ total scores and total acceptability rating scores between VR systems. To compare acceptability ratings across multiple VR systems, a Friedman test was used. A repeated measures correlation was conducted to examine the relationship between baseline and post-VR fatigue reporting.

## Results

**Figure 4.**
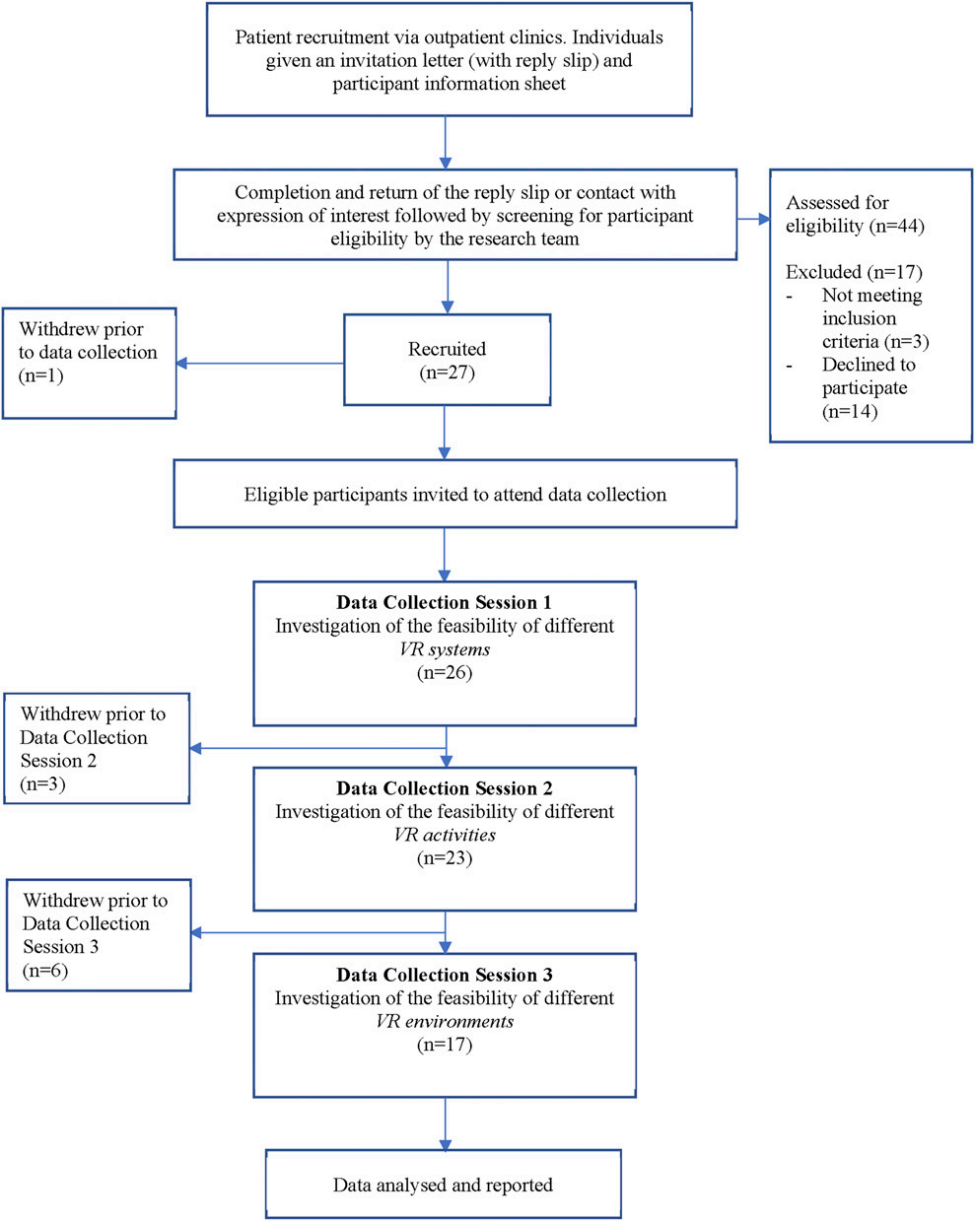
Flow diagram showing overall study flow, processes and participant numbers.

See Figure 4 for a flow diagram showing participant numbers at each phase of the study.

26 participants entered the data collection phase. Patient demographic characteristics were similar between data collection sessions (Table 3) with the majority being female, white British and >45 years of age (mean age at recruitment 48.2 ± 14.3, range 20-72yrs). Most had no previous exposure to VR prior to participation (*n* = 21 out of 26). FMS disease severity ranged from mild to high^34^ (mean FIQR at baseline 65.0 ± 16.2 (moderate severity), total FIQR score range 35.5-92.0).

**Table 3.** Participant characteristics at baseline across the three data collection sessions.

Variable	Number of participants [unless stated otherwise]		
	Session 1: VR systems	Session 2: VR activities	Session 3: VR environments
*n*	26	23	17
BMI (kg/m^2^) [mean (SD)]	29.5 (6.6)	30.0 (6.9)	31.0 (7.3)
Duration of FMS diagnosis (years) [mean (SD)]	5.2 (7.5)	4.3 (5.9)	3.3 (5.0)
FS [mean (SD)]	21.5 (4.9)	21.7 (4.5)	21.7 (4.2)
FIQR [mean (SD)]	65.0 (16.2)	65.4 (16.6)	67.3 (13.9)
PHQ-9 [mean (SD)]	13.7 (5.3)	14.1 (5.5)	13.7 (4.8)
GAD-7 [mean (SD)]	9.5 (5.6)	9.9 (5.6)	9.5 (4.8)
Age			
Age (years) [mean (SD)]	48.2 (14.3)	46.4 (13.8)	46.7 (14.9)
Sex Assigned at birth			
Male:Female ratio	3:23	3:20	2:15
Ethnicity			
White British	24	21	16
Black/African/Caribbean	1	1	1
Other (unspecified)	1	1	0
Previous VR exposure			
No	21	19	14

### Acceptability outcomes

**Figure 5.**
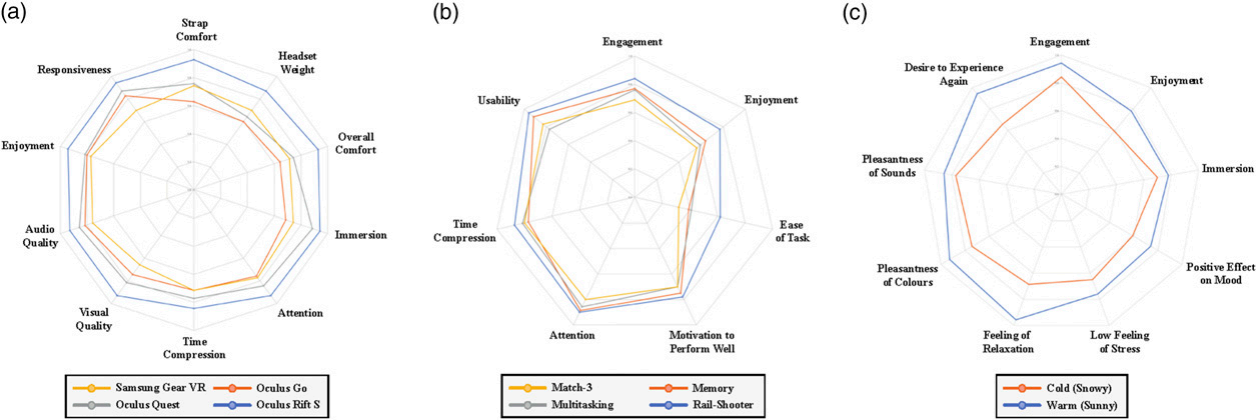
Radar diagram to show acceptability questionnaire scores across the VR systems, activities and environments^a^. ^a^ This diagram includes acceptability data collected through both rating questions (0-7 scale) and virtual VAS. Data has been presented using a standardised scale to aid interpretation (0 = lowest, 1 = highest scores).

See Figure 5 for a radar diagram outlining the key acceptability findings across VR systems, activities and virtual environments.

#### Virtual reality systems

Participants indicated high levels of acceptability across the VR systems when asked to rate their agreement with 15 statements related to system comfort, audio-visual quality, usability (including controllers), and immersion. The mean total scores for these ratings ranged from 77 to 97 out of 105. A comparative analysis between the four VR systems indicated overall differences in reported acceptability (*p* < 0.0001, Friedman test), with the Oculus Rift S being the most acceptable VR system (*p* < 0.0001, Wilcoxon Signed Rank test).

Directly comparing the least acceptable headset (Oculus Go) and most acceptable headset (Oculus Rift S) revealed differences in comfort related to the strap and weight distribution in favour of the Oculus Rift S.

#### Virtual reality activities

Participants reported high levels of acceptability with VR when reporting on their experience with VR generally, across all activities. They rated their agreement with 7 statements enquiring about audio-visual comfort, usability, and engagement within the post-intervention subjective experience questionnaire. The mean total scores for these ratings ranged from 40 to 49 out of 49. Most participants agreed that being presented with a score based on their performance within the VR activities motivated them to perform better (*n* = 19 out of 23).

Participants reported high levels of engagement and immersion across activities, with most agreeing that they had ‘more attention for the activities than for their own thoughts’ and that they ‘lost track of time whilst completing the activities’ (time compression^35^). Upon comparison, the rail-shooter activity was consistently rated the most engaging, user-friendly, and least difficult. In contrast, the match-3 task was rated most difficult and was less favoured in terms of engagement and enjoyment. When accounting for order effect within a mixed effects model, the match-3 task engendered significantly less attention than the rail-shooter task (*p* = 0.028), with no significant differences in time compression.

#### Virtual environments

Participants indicated high levels of acceptability across both virtual environments when asked to rate their agreement with five statements enquiring about audio-visual comfort, engagement and relaxation within the post-intervention subjective experience questionnaire (mean total scores 26-33 out of 35). Greater acceptability across the five statements was seen with the warm (sunny) virtual environment compared to the cold (snowy) environment (*p* = 0.003, *t* test), with superiority also seen in virtual VAS reporting. Most of the participants reported they would prefer to experience the warm virtual environment again in the future (*n* = 14 out of 17).

#### Adverse effects

**Figure 6.**
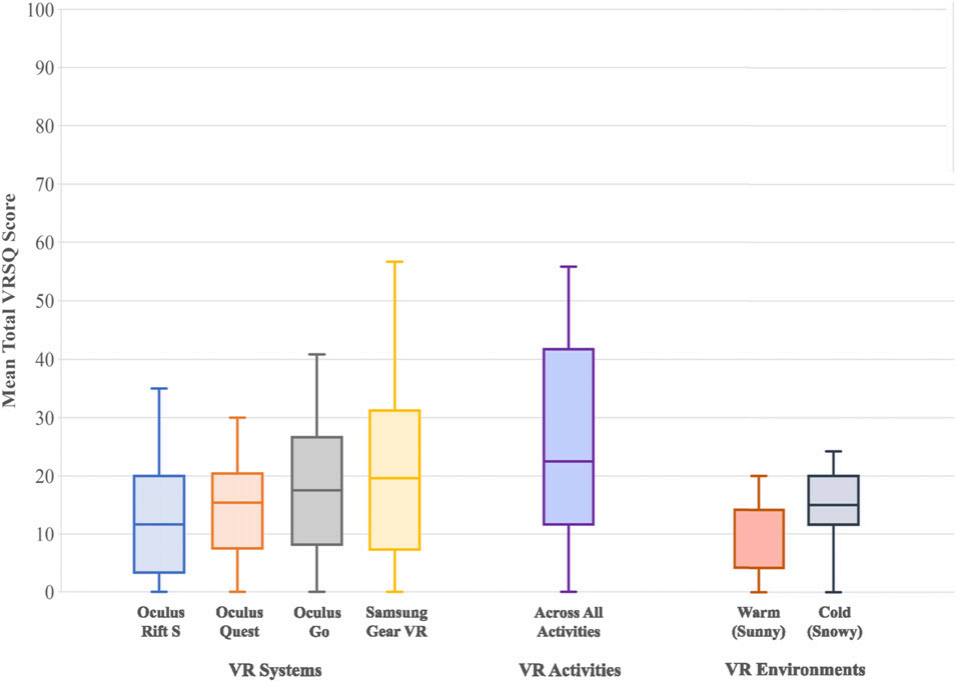
Adverse effects reported following use of each VR system through the Virtual Reality Sickness Questionnaire (VRSQ, maximum total score = 100).

A low rate of adverse effects were reported across VR systems, activities and environments through the VRSQ (Figure 6). The most commonly experienced adverse effects were ‘general discomfort’, ‘fatigue’ and ‘eye strain’, with most being ‘mild’ or ‘moderate’ and settling upon removal of the VR system. ‘Fatigue’ was the most likely adverse effect to persist after removing the VR system. This showed a general increasing trend when accounting for headset order in Data Collection Session 1, suggesting increased reporting with greater durations of VR use. Similarly, adverse effects were more common in Data Collection Session 2 (VR activity comparison), most likely as a consequence of longer durations of continuous VR use (approximately 30 minutes). There was also a positive correlation between baseline fatigue reporting (on the symptom severity scale) and reporting of moderate-severe fatigue post-VR (*n* = 11, r = 0.39).

When comparing VR systems, the Oculus Rift S produced significantly lower adverse effects than the Gear VR and Oculus Go (*p* = 0.013 & 0.018, Wilcoxon Signed Rank test). When asked to specify which VR activities led to adverse effects, similar numbers of participants reported adverse effects with each activity. Fewer adverse effects were reported with the warm compared with the cold virtual environment (VRSQ mean total scores 9.75 ± 10.55 vs 17.94 ± 13.62).

### Other feasibility outcomes

Recruitment numbers are described in Figure 4. 14 individuals declined to participate upon contact, with the majority either feeling too unwell to participate, or conversely too well at the time of recruitment with minimal pain. Overall, 10 out of 26 participants dropped out of the study, with one participant dropping out before data collection commenced, three participants after Data Collection Session 1, and six participants after Data Collection Session 2. Reasons for drop out included ill health (*n* = 2), lack of time or practical difficulties with attending sessions (*n* = 2), inability to contact (*n* = 4), and other (*n* = 2). No participants stated their reasons for dropping out were due to adverse effects experienced with VR. All questionnaires were fully completed by participants, resulting in no missing data for the study.

Following use of all VR systems, participants reported their openness to using VR in the future at home and as part of their pain treatments. Assuming that VR was as effective as the participants’ current method for managing pain and was made freely available, all of the participants agreed that they would be ‘open to using VR technology at home regularly for the management of [their] pain’ and most agreed that they could ‘see a future for the use of VR as a pain management option’ following completion of Data Collection Session 1 (*n* = 23 out of 26).

### Clinical outcomes

Clinical outcome data relating to pain and mood are shown in the Appendix.

Reductions in both mean pain VAS and MPQ-SF total scores were reported across all VR systems, activities and virtual environments. In Data Collection Session 1, the Oculus Rift S produced the greatest pain reduction, and the Oculus Go the least. In Data Collection Session 3, the warm (sunny) virtual environment produced the greatest pain reduction, and the cold (snowy) environment the least. There was considerable variability in VAS reporting between participants after interventions. For example, one participant reported a reduction in VAS from 7.3 (baseline) to 1.4 (post-intervention) after using all VR systems in Data Collection Session 1, whereas another reported a small increase in VAS.

After experiencing all VR activities in Data Collection Session 2, the majority of participants reported either improvements or no change in their mood, with only small differences between activities.

In Data Collection Session 3, participants reported low subjective feelings of stress and strong feelings of relaxation with both virtual environments on the virtual VAS, with preference for the warm (sunny) environment (Figure 5). A considerably greater number of participants reported improvements in mood after experiencing the warm (sunny) virtual environment (*n* = 16 out of 17) compared with the cold (snowy) environment (*n* = 4 out of 17).

## Discussion

### Study overview and context

This study is the first to specifically investigate the feasibility of different head-mounted VR systems, environments and activities in patients with FMS. Across this multi-faceted study, the findings indicated that interactive VR designed to engage attention had good feasibility in patients with FMS, as shown by high levels of acceptability, low incidence of adverse effects, and positive perceptions. In common with previous VR pain studies, most patients with FMS felt interactive VR activities were highly immersive and effective at engaging attention. These findings provide additional evidence regarding the feasibility of using VR as a future digital therapeutic in FMS and provide information to inform future larger-scale studies.

This study builds on previous VR research within non-specific chronic pain^17,36^ and chronic low back pain.^19,20^ The recent longitudinal randomised controlled trials investigating VR interventions in low back pain demonstrate the efficacy and utility of VR as a home-based non-pharmacological treatment, leading to one application (RelieVRx) gaining approval from the Food and Drug Administration (FDA). However, given people living with FMS have specific challenges which would likely influence their experience of VR, studies in this cohort are essential. The few studies investigating ‘virtual reality’ specifically in FMS have mostly used non-immersive (not head-mounted) systems such as 2D projection screens.^37^ These studies were not designed to investigate modern, head-mounted, interactive VR interventions aiming to engage attention. Similarly, most therapeutic VR studies evaluate one VR system, activity and virtual environment without considering the impact of each component.

### Challenges and considerations with VR system components

VR interventions are ‘complex’ given they have multiple interacting components that each can influence patient outcomes.^38,39^ The strength of this study lies in its multi-faceted approach to investigate each of the important components that make up an attention-based VR intervention. Despite positive acceptability reported across VR systems, it is clear from this study that suboptimal audio-visual quality, latency, strap comfort and weight distribution can adversely affect feelings of immersion, engagement of attention and overall enjoyment in FMS. Consequently, these system-specific features can lead to greater adverse effects, and the possibility that this could impact clinical outcomes should not be ignored. Although the VR systems used in this study are now outdated by newer, more technologically advanced versions, there remains considerable variation in available devices with more customisation options than even before. This study’s findings are particularly important in this context, as they highlight the importance of optimising comfort through carefully considered VR system setup to enhance user experience and minimise adverse effects. Clear and systematic reporting of such adverse effects is essential yet remains inconsistent across VR studies. To address this, future research should prioritise the development of standardised protocols for assessing and reporting adverse effects to ensure a comprehensive understanding of safety, especially for populations with specific needs like those with FMS.

### Significance of the virtual environment

Study findings also demonstrated the importance of virtual environment design when creating VR content for patients with FMS. Perhaps as a consequence of the early work conducted by Hoffman et al utilising cold virtual environments for patients with burns injuries,^16^ other studies have also used cold environments for chronic pain.^17^ In contrast, our findings indicate improved acceptability related to virtual environments simulating warm conditions in FMS. This may be disease-specific, given the unique sensory hypersensitivity and thermodysregulatory states seen in FMS. It may also be an indication of a more complex relationship informed by a person’s pain characteristics or previous experiences, indicating the need to tailor the virtual environment to the user. Qualitative responses perhaps explain differences in reported acceptability, with participants indicating an association with relaxation in warm settings and a perceived worsening of symptoms in cold settings. This is also shown in our mood data, with the majority indicating an improvement in their mood with the warm virtual environment. Although no studies have specifically explored the role of patients’ phobias or past experiences in this context within relevant chronic pain populations, this is an important consideration that could influence the acceptability and safety of VR interventions. Regardless of the mechanism, it is clear that the virtual environment is a crucial element in VR chronic pain therapeutics. Further research should investigate a range of environments in well-characterised pain cohorts and consider evaluating factors like phobias or past experiences before starting VR therapy to better tailor environments to individual needs.

### The importance of fatigue with VR

This study’s findings provide reassurance of the safety of using VR in FMS, with low adverse effects reported across VR systems, activities and environments. However, a general increase in fatigue reporting with longer VR sessions was noted, particularly apparent during Data Collection Session 2 where VR use duration was at its peak. Upon closer examination, there was a relationship between baseline fatigue levels and subsequent fatigue reporting with VR use. Considering that fatigue is a defining characteristic of FMS and other chronic pain conditions, these indirect findings underscore the importance of customising VR ‘dosing' for high-risk groups. This includes integrating safety measures such as ‘pacing checkpoints’ and conducting more targeted research to understand the ‘dose-response’ relationship of VR in specific disease subgroups.

### Perceptions and tailored VR interventions

Prior to this study, the perceptions of individuals with FMS regarding VR use for pain management were unclear. Although only within a small, select sample of patients, study findings demonstrated positive attitudes towards the use of VR for future, home-based therapy.

The rapid evolution of VR in recent years, particularly with the advent of affordable and portable systems, has opened new avenues for the home-based management of chronic pain. With the increasing availability of VR, it is inevitable that the use of VR therapeutics will become more widespread. Our data in FMS highlights the risk of adopting a generalised approach with use of VR applications across disease subtypes that are unsuitable and may lead to adverse effects.

To ensure the acceptability and safety of VR interventions, future research and application development should promote: (1) co-design with individuals who have lived experience of chronic pain, (2) consideration of each VR component’s impact on acceptability, tolerability, and usability, and (3) evaluation in specific chronic pain subgroups, taking into account unique disease-specific challenges. This approach will help tailor VR applications to meet the diverse needs of chronic pain patients.

### Limitations and recommendations for future research

Limitations of the present study need to be acknowledged. The study was primarily designed to determine feasibility, with a small sample size appropriate for the study design. While the study sample was representative of the UK FMS population,^40^ the inclusion of mostly white females based in the East of England limits the generalisability of findings. Measurement of outcomes at multiple time points provided a good understanding of intervention feasibility and helped reduce recall bias by collecting relevant data for each intervention as close to the intervention completion as possible. Despite this, use of self-report questionnaires risks recall bias, particularly with questionnaires comparing interventions at the end of the session. The use of non-validated subjective experience questionnaires and virtual scales may limit the generalisability and robustness of the findings. These tools were specifically developed for each data collection session to gather targeted feedback on comfort, usability, and overall user experience. Available validated scales were either not specific for VR interventions, or evaluated single constructs like immersion and lacked the specificity needed for assessing factors such as the comfort of individual VR system components.^41^ Data on the ease of completion of the questionnaires for participants or of data analysis for researchers was not collected. The sequential nature of the data collection sessions in this study leads to the possibility of intervention novelty influencing outcomes. This should be taken into account for future studies. The one-week washout period aimed to minimise potential carryover effects between sessions. However, due to limited knowledge about the duration of effects from attention-based VR, some risk of residual influence remains. Future trials should account for this uncertainty in their design to ensure accurate interpretation of outcomes.

While the absence of a control intervention is not a specific limitation for our feasibility study, it is an important consideration for future VR trials evaluating effect. The VR-CORE international working group emphasise the lack of a ‘perfect’ or ‘standardised’ control condition for VR therapeutics research, with the choice of control depending on the research question.^21^ Our feasibility study focused on evaluating short-term outcomes, but future studies should consider longer follow-up to gain insights into the sustained feasibility of VR use, including the persistence of benefits or potential delayed adverse effects with extended use.

The overall attrition rate of 37.0% resulted in complete datasets for 16 participants, falling short of our target sample size of 20-30. Multiple data collection sessions organised over time may have increased the risk of participant dropout, especially given the fluctuating nature of symptoms in individuals with FMS. While participants did not cite COVID-19 as a direct reason for dropout, many communicated difficulties with transport and increased anxieties related to social contact during the pandemic. Additionally, national lockdowns required us to pause data collection, extending the data collection period to 10 months, which may have further influenced retention. These factors should be considered when interpreting our attrition rate, as they represent barriers that may not be as prevalent in future studies. Despite these challenges, the high rate of questionnaire completion by those who attended suggests that the outcome measures were not overly burdensome and could be suitable for future research. To account for attrition in similar studies, a larger initial sample size could help ensure that sufficient data are collected. Future trials should carefully consider using multiple lab-based data collection sessions over time, and may also consider remote or hybrid data collection methods to reduce barriers to participation.

### Conclusion

In conclusion, this study increases confidence with respect to the feasibility of using attention-based, head-mounted VR interventions in patients with FMS. Strong acceptability, minimal adverse effects and positive perceptions of using VR highlight the potential of this technology to support future home-based, non-pharmacological treatments in FMS. However, it is clear that VR interventions are complex with the need to consider the acceptability of each component of the VR system, environment and activity when attempting to improve clinical outcomes. It is therefore essential that we avoid a ‘one-size-fits-all’ approach to future VR therapeutics, and instead promote co-development and tailoring of interventions to the patients that they are designed for.

### Supplemental Material

Supplemental Material - Feasibility of attention-based virtual reality interventions in fibromyalgia syndrome: comparing systems, virtual environments and activities Supplemental Material for Feasibility of attention-based virtual reality interventions in fibromyalgia syndrome: comparing systems, virtual environments and activities by Jordan Tsigarides, Vanessa Grove, Jacqueline Chipping, Jack Dainty, Susan Miles, Nicholas Shenker, Saber Sami and Alexander Macgregor in British Journal of Pain

Supplemental Material - Feasibility of attention-based virtual reality interventions in fibromyalgia syndrome: comparing systems, virtual environments and activities Supplemental Material for Feasibility of attention-based virtual reality interventions in fibromyalgia syndrome: comparing systems, virtual environments and activities by Jordan Tsigarides, Vanessa Grove, Jacqueline Chipping, Jack Dainty, Susan Miles, Nicholas Shenker, Saber Sami and Alexander Macgregor in British Journal of Pain

Supplemental Material - Feasibility of attention-based virtual reality interventions in fibromyalgia syndrome: comparing systems, virtual environments and activities Supplemental Material for Feasibility of attention-based virtual reality interventions in fibromyalgia syndrome: comparing systems, virtual environments and activities by Jordan Tsigarides, Vanessa Grove, Jacqueline Chipping, Jack Dainty, Susan Miles, Nicholas Shenker, Saber Sami and Alexander Macgregor in British Journal of Pain
