# Supplementary material for: Feasibility of attention-based virtual reality interventions in fibromyalgia syndrome: comparing systems, virtual environments and activities
Source: Br J Pain. 2025 Jan 4;19(2):71–85. doi: 10.1177/20494637241310696 (PMC11700397; doi:10.1177/20494637241310696)
Supplement: Supplemental Material - Feasibility of attention-based virtual reality interventions in fibromyalgia syndrome: comparing systems, virtual environments and activities [file sj-pdf-1-bjp-10.1177_20494637241310696.pdf]

Title & Description: Appendix 1 – Specifications of the VR Application and Systems Used

	**VR System**			
	**Samsung Gear VR**	**Oculus Go**	**Oculus Quest**	**Oculus Rift S**
**Image**	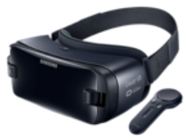	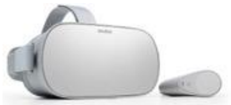	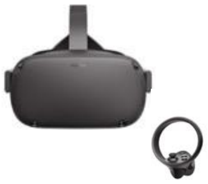	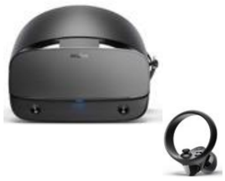
**Internal Chipset**	Exynos 8895	Qualcomm Snapdragon 821	Qualcomm Snapdragon 835	N/A[a]
**Random-access Memory** (RAM)	4gb	3gb	4gb	N/A[a]
**Screen Resolution** (per eye)	1280x1440	1280x1440	1440x1600	1280x1440
**Screen refresh rate**	60hz	72hz	72hz	80hz
**Weight**	500g	468g	571g	563g

Table 4: Comparison of the VR system specifications.

[a] A Dell Mobile Precision 7740 laptop (Windows 10 Pro, Intel Core i9 processor, 64gb DDR4 ram, Nvidia Quadro RTX 5000 16gb GDDR6) streamed VR content to the Rift S, tethered through a USB-Display Port cable

The VR Application Technical Specifications

The application was built and tested using Unity 2019.4. It was designed to be stereoscopic with the ability for the user to perceive depth and three-dimensional structures. The untethered systems (Gear VR, Oculus Go and Oculus Quest) used an Android build, whereas the tethered Oculus Rift S used a Windows™ build. Data collected from 'virtual' VR questionnaires and on program use (including timestamped data on environment/activity selection and activity performance) were stored within a unique JSON file written directly to

the study computer. This data was only recorded during Data Collection Sessions 2 and 3 when using the Oculus Rift S (tethered to the study laptop).

Custom head mounting straps were used with the Oculus Go and disposable fascia covers used with the Gear VR to enable the hygiene protocol to be completed.

The VR Activities Description

Rail-Shooter Activity – A ‘rail shooter’ activity requiring hand-eye co-ordination and agility. Travelling slowly aboard a boat down a calm river, the user is presented a variety of targets that they are required to aim at and hit. There are two different target types, with some moving and some remaining static. A score was earned when hitting targets, with higher scores provided with hits closer to the centre of the target.

Memory Activity – A sequence recall activity requiring short-term memory and spatial awareness. A 3x3 grid of soil plots presents a sequence of emerging plants to the user who has to replicate the sequence. This sequence escalates in length (starting at 3), adding one extra to the sequence each time the user correctly replicates the preceding sequence until failure. Upon the first instance of an incorrect selection, the user is given a warning prior to the sequence resetting back to 3 if they select the incorrect plot on two occasions sequentially. The user gained a score equal to the length of the sequence upon correctly recalling and replicating the sequence.

Multitasking Activity – A multitasking activity requiring working memory and reasoning skills. A patch of soil with five plots, a watering can and seed container are presented to the user. The user is challenged to follow a sequence of four tasks (digging a plot, planting a seed, watering and harvesting) for each of the five plots in order to grow trees. Each harvested tree provided a score for the user, with the requirement to manage multiple plots simultaneously.

Match-3 Activity – A ‘match three’ activity requiring visual searching, visuospatial awareness and problem-solving skills. Three vines with multiple vegetables attached are presented to the user. Pulling each vine downwards or upwards changed the position of the vegetable and revealed a new vegetable from above or below (depending on the direction the vine is pulled). When the user matched three identical vegetables in a horizontal row they would gain a positive score. An escalating ‘multiplier’ was gained for successful matching of three items in quick succession, improving the overall score gained.
