# Supplementary material for: Feasibility of attention-based virtual reality interventions in fibromyalgia syndrome: comparing systems, virtual environments and activities
Source: Br J Pain. 2025 Jan 4;19(2):71–85. doi: 10.1177/20494637241310696 (PMC11700397; doi:10.1177/20494637241310696)
Supplement: Supplemental Material - Feasibility of attention-based virtual reality interventions in fibromyalgia syndrome: comparing systems, virtual environments and activities [file sj-pdf-2-bjp-10.1177_20494637241310696.pdf]

Title & Description: Appendix 2 – Detailed Description of the Outcomes Measures Used

Disease Severity Measures (Baseline)

Widespread Pain Index (WPI) and Symptom Severity Scale (SSS) Scores:

The Widespread Pain Index (WPI) is a questionnaire enquiring about pain experienced across 19 body areas, to evaluate the presence and distribution of pain experienced by the patient over the past week.

The Symptom Severity Scale (SSS) is a questionnaire with two parts: the first part includes 13 questions assessing the severity of fatigue, waking unrefreshed, cognitive symptoms, and the extent of somatic symptoms in general, while the second part evaluates the number of somatic symptoms experienced.

The sum of the Widespread Pain Index (WPI) and Symptom Severity Scale (SSS) scores are used to calculate the Fibromyalgia Severity (FS) Score to categorise FMS symptom severity (FS, total score 0-31).

Revised Fibromyalgia Impact Questionnaire (Revised FIQR):

Self-report questionnaire to determine impact of FMS on activities of daily living, overall wellbeing and severity of symptoms. 21 questions rated on a 0-10 numeric rating scale (NRS) assessing the impact of FMS on three domains (1) function, (2) overall impact, and (3) symptoms. The total FIQR score is the sum of the three domain scores (0-100).

Depression and Anxiety Measures (Baseline)

Patient Health Questionnaire 9 (PHQ-9):

Self-report questionnaire to determine the presence and severity of depressive symptoms. 9 items each scored from 0 (not at all) to 3 (nearly every day). Total scores range between 0-27.

Generalised Anxiety Disorder Assessment 7 (GAD-7):

Self-report questionnaire to determine the presence and severity of anxiety symptoms. 7 items scored from 0 (not at all) to 3 (nearly every day). Total scores range between 0-21.

Subjective Experience Questionnaires (Post-Intervention)

These questionnaires included a combination of rating questions, asking participants to rate their level of agreement with statements related to acceptability on a scale of 1-7 (1 strongly disagree, 7 strongly agree), multiple choice questions, white space questions (to contextualise quantitative self-report), and virtual VAS ratings (10-point blinded scale). Questionnaires varied between data collection sessions and were tailored to the intervention:

1. VR Systems:
    - After use of a VR system: 15 rating questions and 3 white space questions.
    - After use of all VR systems: 6 multiple choice questions (asking the participant to choose the best performing headset for a variety of acceptability questions) and 3 rating questions enquiring about perceptions of VR.
2. VR Activities:
    - After experiencing a VR activity: 5 virtual VAS rating questions enquiring about enjoyment, engagement, difficulty, length of time in activity and motivation to perform the activity.

- After experiencing all VR activities: 8 initial rating questions (generic acceptability across the activities), followed by 3 further rating questions enquiring about perceptions of VR. This was followed by 20 rating questions (5 per activity, activity-specific acceptability), 8 multiple choice questions (asking about specific effects on pain and mood for each activity) and 12 white space questions (3 per activity).

3. VR Environments:
    - After experiencing a VR environment: 4 virtual VAS rating questions enquiring about enjoyment, immersion, feelings of stress and changes in mood related to the virtual environment.
    - After experiencing both VR environments: 2 multiple choice questions asking participants about the effect of each environment on pain and mood with 2 associated white space questions. 10 initial rating questions (5 per environment) enquiring about acceptability of the colours and sounds, in addition to engagement, feelings of relaxation and desire to experience the environment again in the future. 1 further multiple choice question asking the participant to choose which one environment they would rather experience again with white space questions to describe likes and dislikes of each. 3 further rating questions enquiring about perceptions of VR.

The Virtual Reality Sickness Questionnaire (VRSQ, Post-intervention)

Participants completed the VRSQ after use of each VR headset in Data Collection Session 1 and after experiencing all environments/activities in sessions 2 and 3. Participants were asked to rate the severity of each symptom on a four-point scale ranging from 'None' (0) to 'Severe'

(3). VRSQ scores were calculated by first summing the scores for each symptom in the two categories. These raw scores were then weighted according to the procedure outlined by Kim et al. (2018): oculomotor score was the sum of raw scores divided by 12 and then multiplied by 100, and disorientation was divided by 15 and multiplied by 100. The total VRSQ score was computed as the sum of the oculomotor and disorientation scores divided by 2. The VRSQ allowed assessment of both the incidence (i.e., the number of participants reporting symptoms) and severity (i.e., the intensity of symptoms reported) of adverse effects post-VR.

Pain Intensity (Pre/Post Intervention)

To quantify participants' perception of pain, we implemented a multimodal approach using the McGill Pain Questionnaire Short Form (MPQ-SF), a Visual Analog Scale (VAS), and a custom post-intervention questionnaire.

The MPQ-SF, a brief yet robust tool for pain assessment, was administered to all participants both before (pre-exposure baseline) and after (post-exposure) intervention. This instrument includes 15 pain descriptors (11 sensory; 4 affective), which participants rate based on their intensity from 0 (none) to 3 (severe). The MPQ-SF yields three scores: the sensory, affective, and total pain scores, providing a multidimensional representation of the pain experience.

The VAS, a simple and reliable tool for subjective pain measurement, was also employed. Two forms of the VAS were used, a physical paper-based version and a ‘virtual’ version. For the written VAS, participants were asked to rate their pain intensity on a 10cm line, ranging from "no pain" on one end (0cm) to "worst possible pain" on the other end (10cm). The ‘virtual’ VAS replicated the written VAS but used a 10-point blinded scale (participants were only able to appreciate the position of a marker on a line, without labelling of numbers) with data recorded by the application. The ‘virtual’ pain VAS was completed in VR after

experiencing each environment and activity during Studies 2 and 3. The written pain VAS was completed at baseline and post-intervention in all studies, as well as after use of each VR system in Study 1. Recording of VAS pre- and post-intervention, and between multiple interventions, allowed for a direct comparison of pain levels at different time points.

Repeated measures correlation analysis demonstrates a correlation coefficient of 0.403 (p<001) when assessing the correlation of virtual and written VAS reporting in Data Collection Session 2. In line with work by Boonstra et al (2014), interpretation of VAS scores are reported as follows: mild pain (≤3.4cm), moderate pain (3.5-7.4cm), and severe pain (≥7.5cm).

In addition to the MPQ and VAS, we included questions within the subjective experience questionnaires to gain information about participants' perception of pain in relation to specific interventions. Participants indicated whether each intervention increased, decreased, or did not change their perception of pain using a multiple-choice format. Additionally, participants were asked to complete white space questions to explain why they felt their pain levels were impacted.
