# Supplementary material for: Feasibility of attention-based virtual reality interventions in fibromyalgia syndrome: comparing systems, virtual environments and activities
Source: Br J Pain. 2025 Jan 4;19(2):71–85. doi: 10.1177/20494637241310696 (PMC11700397; doi:10.1177/20494637241310696)
Supplement: Supplemental Material - Feasibility of attention-based virtual reality interventions in fibromyalgia syndrome: comparing systems, virtual environments and activities [file sj-pdf-3-bjp-10.1177_20494637241310696.pdf]

Title & Description: Appendix 3 – Clinical Outcome Data Tables

Pain reporting

Reporting Time Point	Pain VAS [mean (SD)]	Difference in VAS from Session Baseline [mean (95% CIs)]	Number achieving MCID in VAS	MPQ-SF total score [mean (SD)]	Difference in MPQ-SF from Session Baseline [mean (95% CIs)]
Data Collection Session 1 (VR Systems, n=26)					
Session 1 Baseline	5.5 (1.9)	-	-	24.3 (9.0)	-
Post-VR System: Samsung Gear VR	4.7 (1.8)	-0.8 (-1.3, -0.4)	12	-	-
Post-VR System: Oculus Go	4.9 (1.8)	-0.6 (-1.2, -0.1)	8	-	-
Post-VR System: Oculus Quest	4.3 (1.6)	-1.2 (-1.9, -0.5)	13	-	-
Post-VR System: Oculus Rift S	3.9 (1.8)	-1.6 (-2.3, -0.9)	19	-	-
Post-Intervention (following use of all VR systems)	4.0 (1.9)	-1.5 (-2.2, -0.8)	16	14.6 (9.1)	-9.7 (-13.0, -6.4)
Data Collection Session 2 (VR Activities, n=23)					
Session 2 Baseline	5.5 (2.3)	-	-	21.7 (8.8)	-
Post-Activity: Rail-shooter	4.1 (2.4)	-1.4 (-2.1, -0.6)	14	-	-
Post-Activity: Memory	4.0 (2.4)	-1.5 (-2.2, -0.7)	13	-	-
Post-Activity: Multitasking	4.3 (2.4)	-1.2 (-2.0, -0.4)	13	-	-
Post-Activity: Match-3	4.0 (2.2)	-1.5 (-2.1, -0.8)	14	-	-

Post-Intervention: After Experiencing All Activities	4.5 (2.5)	-1.0 (-1.9, -0.1)	14	16.9 (10.5)	-4.8 (-7.4, -2.2)
Data Collection Session 3 (VR Environments, n=17)					
Session 3 Baseline	5.4 (2.1)	-	-	21.6 (10.1)	-
Post-environment: Warm (sunny)	4.2 (2.0)	-1.2 (-2.1, -0.4)	11	-	-
Post-environment: Cold (snowy)	4.6 (2.2)	-0.8 (-1.5, -0.1)	6	-	-
Post-Intervention: After Experiencing Both Environments	4.8 (2.5)	-0.6 (-1.4, 0.2)	8	15.2 (9.1)	-6.4 (-9.0, -3.8)

Table 5: Comparison of pain outcomes across the study. Minimal Clinically Important Difference (MCID) is defined as a reduction in VAS by ≥15% (as described by Salaffi et al in musculoskeletal disease[1])

Mood Reporting

**Type of Activity or Environment**	**Post-intervention: Number Reporting Improvement in Mood**	**Post-intervention: Number Reporting Worsening in Mood**
Data Collection Session 2 (VR Activities, n=23)		
Rail-shooter	10	1
Memory	9	4
Multitasking	9	4
Match-3	5	4
Data Collection Session 3		

(VR Environments, n=17)		
Warm (Sunny)	16	0
Cold (Snowy)	4	1

Table 6: Comparison of mood-related outcome measures across the VR activities and environments.

REFERENCES

1. Salaffi F, Stancati A, Silvestri CA, et al. Minimal clinically important changes in chronic musculoskeletal pain intensity measured on a numerical rating scale. *Eur J Pain* 2004; 8: 283-291. DOI: 10.1016/j.ejpain.2003.09.004.
